# Chemical–protein interaction extraction via contextualized word representations and multihead attention

**DOI:** 10.1093/database/baz054

**Published:** 2019-05-24

**Authors:** Yijia Zhang, Hongfei Lin, Zhihao Yang, Jian Wang, Yuanyuan Sun

**Affiliations:** College of Computer Science and Technology, Dalian University of Technology, Dalian, China

## Abstract

A rich source of chemical–protein interactions (CPIs) is locked in the exponentially growing biomedical literature. Automatic extraction of CPIs is a crucial task in biomedical natural language processing (NLP), which has great benefits for pharmacological and clinical research. Deep context representation and multihead attention are recent developments in deep learning and have shown their potential in some NLP tasks. Unlike traditional word embedding, deep context representation has the ability to generate comprehensive sentence representation based on the sentence context. The multihead attention mechanism can effectively learn the important features from different heads and emphasize the relatively important features. Integrating deep context representation and multihead attention with a neural network-based model may improve CPI extraction. We present a deep neural model for CPI extraction based on deep context representation and multihead attention. Our model mainly consists of the following three parts: a deep context representation layer, a bidirectional long short-term memory networks (Bi-LSTMs) layer and a multihead attention layer. The deep context representation is employed to provide more comprehensive feature input for Bi-LSTMs. The multihead attention can effectively emphasize the important part of the Bi-LSTMs output. We evaluated our method on the public ChemProt corpus. These experimental results show that both deep context representation and multihead attention are helpful in CPI extraction. Our method can compete with other state-of-the-art methods on ChemProt corpus.

## Introduction

Accurately detecting the interactions between chemicals and proteins is a crucial task that plays a key role in precision medicine, drug discovery and basic clinical research (1). Currently, PubMed contains >28 million articles, and its annual growth rate is more than a million articles each year. A large amount of valuable chemical–protein interactions (CPIs) are hidden in the biomedical literature. There is an increasing interest in CPI extraction from the biomedical literature.

Since manually extracting biomedical relations such as protein–protein interactions (PPI) and drug–drug interactions (DDI) is costly and time-consuming, some computational methods (2–6) have been successfully proposed for automatic biomedical relation extraction. For example, Kim *et al.* (4) proposed using a subsequence kernel for PPI extraction that matches the e-walk and v-walk on the shortest dependency to capture the noncontiguous syntactic structures. Segura-Bedmar *et al.* (7) employed linguistic patterns to extract DDIs. Currently, models based on deep neural networks have exhibited surprising potential in biomedical relation extraction (8–10). Rois *et al.* (11) proposed an adversarial domain adaptation method to extract PPIs and DDIs. Zhang *et al.* (12) proposed a hybrid deep neural model for biomedical relation extraction from the biomedical literature, which integrates the advantages of convolutional neural networks (CNNs) and recurrent neural networks (RNNs).

To date, most studies on the biomedical relation extraction have focused on the PPIs and DDIs, but a few attempts have been made to extract CPIs. The BioCreative VI ChemProt shared task (13) released the ChemProt dataset for CPI extraction, which is the first challenge for extracting CPIs. The ChemProt dataset (13) provided an opportunity to compare current CPI extraction methods on the same benchmark corpora. Peng *et al.* (14) proposed an ensemble method to integrate the support vector machines (SVMs) and deep neural networks and achieved an *F*-score of 0.641 on the ChemProt dataset. Corbett and Boyle (15) employed transfer learning and specialized word embeddings to extract CPIs and achieved an *F*-score of 0.615 on the ChemProt dataset.

From the BioCreative VI ChemProt track, neural network-based methods achieved state-of-the-art performance in CPI extraction. Compared with feature-based and kernel-based methods, the deep neural networks can automatically learn latent features. So far, the best performance of CPI extraction is an *F*-score of 0.641 on the ChemProt corpus (14). One of the bottlenecks of neural networks in natural language processing (NLP) is word embeddings that are generally the input layer of various neural networks. Pretrained word embeddings (16, 17) are of great importance for the performance of neural network-based methods in NLP tasks. Learning high-quality distributed word embeddings is very challenging. Although great efforts have been made in distributed word representations, current word embeddings still cannot effectively vary across linguistic contexts. Most recently, Peters *et al.* (18) proposed deep contextualized word representations called ELMo based on a deep bidirectional language model. Traditional word embeddings represent each token as a unique embedding vector. However, ELMo represents each token as a function of the entire input sentence, which makes the representation of each token dependent on the sentence context. Therefore, integrating the ELMo representation with deep neural networks can provide more comprehensive input representation for the following neural network models and may improve the performance of CPI extraction.

Another challenge in CPI extraction is how to accurately detect and extract the CPIs in long and complicated sentences. In particular, the chemical and protein entities are often found in different clauses. It is hard to capture the distinguished syntactic information for deep neural networks in these long and complicated sentences. Recent studies (19, 20) have suggested attention mechanisms can effectively emphasize the relatively important parts of the input sentences and be helpful in boosting the performance of relation extraction. However, most studies only employed single attention in the deep neural models. Multihead attention applies attention multiple times and divides attention information into multiple heads (21). Thus, a multihead attention mechanism will make it easier to capture the relevant important information for deep neural networks in CPI extraction.

In this work, we explore the effectiveness of deep contextualized word representations and multihead self-attention mechanisms in the CPI extraction. We introduce a deep neural model to extract CPIs from the literature, which includes an ELMo input layer, bidirectional long short-term memory networks (Bi-LSTMs) and a multihead attention layer. Liu *et al.* (22) integrated attention pooling into the gated recurrent unit (GRU) model to extract CPIs. Verga *et al.* (23) combined the multihead attention with CNNs to construct transformer model to extract the document-level biomedical relations. In this work, we combined the multihead attention with Bi-LSTMs. In particular, we employed the ELMo contextualized representation in the input layer. To the best of our knowledge, this is the first model that used ELMo contextualized representation for biomedical relation extraction. Our proposed model is evaluated on the ChemProt corpus. The experimental results show that both contextualized word representations and multihead attention are valuable for CPI extraction. Our model can effectively integrate the contextualized word representations and multihead attention for CPI extraction and achieve state-of-the-art performance on ChemProt corpus. We have also shown that our model can also achieve competitive performance on other biomedical relation extraction tasks such as DDI extraction.

**Figure 1:**
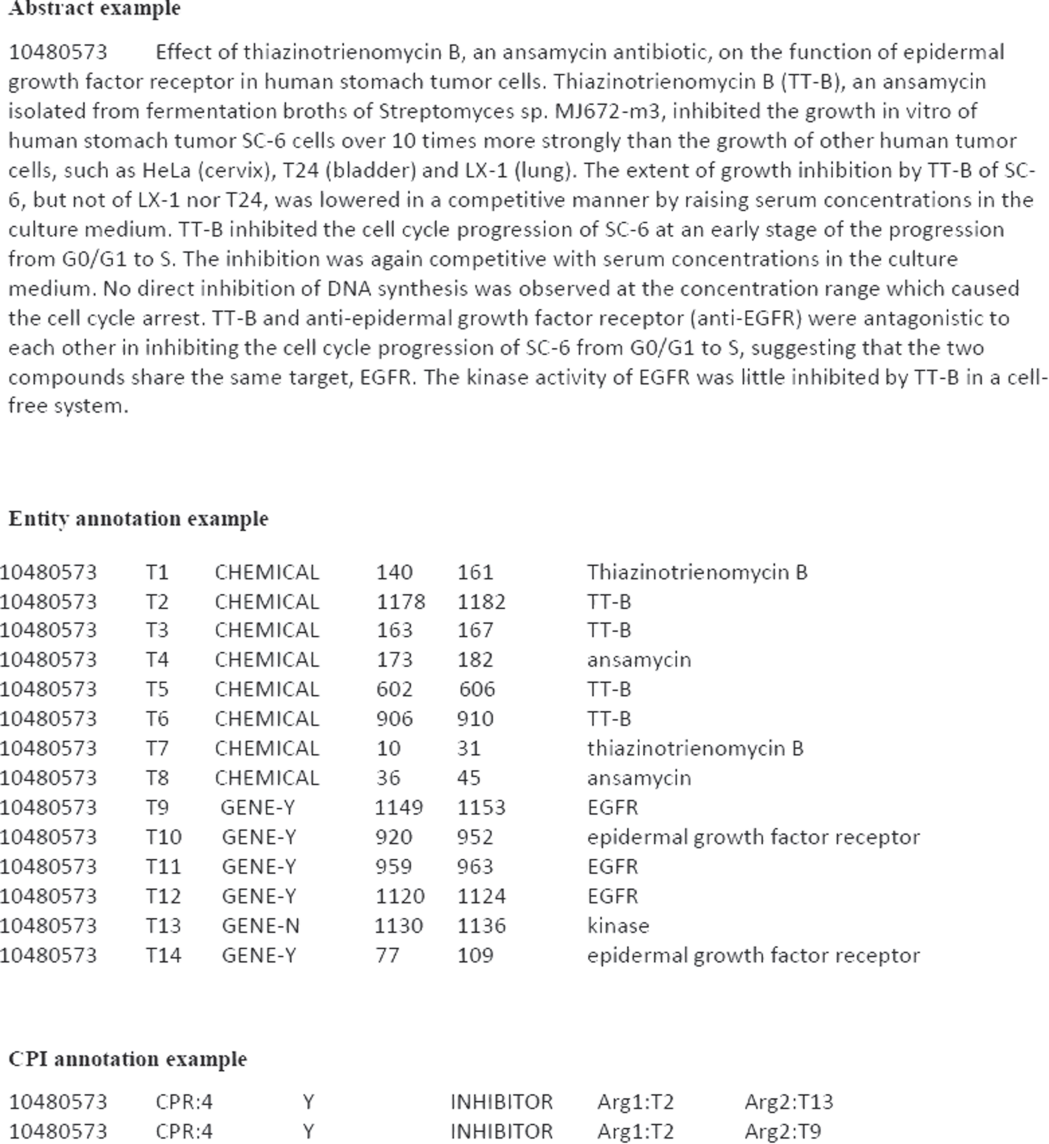
The illustrative examples of ChemProt corpus.

## Materials and methods

### CPI extraction

Computational CPI extraction is generally approached as a task of classifying whether a specified semantic relation holds between the chemical and protein entities within a sentence or document. The ChemProt corpus is a manually annotated CPI dataset, which greatly promotes the development of CPI extraction approaches. The ChemProt corpus includes training, development and test sets. Each set contains the corpus file, entity annotation file and relation annotation file. Figure 1 gives an example of the corpus file, entity annotation file and relation annotation file from the ChemProt training set. The corpus file gives the abstract document and the PubMed Unique Identifier (PMID) (10480573). The entity file gives all chemical and gene/protein entity mentions in the abstract. The entity annotation information includes the PMID, entity number, type of entity mention (‘CHEMICAL’, ‘GENE-Y’ and ‘GENE-N’), start and end character offset of the entity mention and text string of entity mention. ‘GENE-Y’ denotes the entity mention can be associated with a biological database identifier. ‘GENE-N’ denotes the entity mention cannot be associated with a biological database identifier. The relation file gives the detailed CPI annotations in the abstract. It contains PMID, CPI relation class, evaluation type (Y: evaluated; N: not evaluated), CPI relation and interactor arguments. In Figure 1, it can be seen that 14 chemical or gene/protein entities and 2 CPIs are annotated in the abstract (PMID: 10480573). In the BioCreative VI ChemProt share task, the named entity recognition has been already done, and the participant teams only focused on CPI extraction.

Table 1 shows the 10-type relation classes of the ChemProt corpus. In Table 1, it can be seen that each relation class includes one or multiple relation types. ‘Eval.’ denotes whether the relation class is evaluated in the BioCreative VI ChemProt share task. Although the ChemProt corpus contains 10-type relation classes, only 5-type relation classes were evaluated, including CPR:3, CPR:4, CPR:5, CPR:6 and CPR:9. Table 2 shows the statistics of the ChemProt corpus.

**Table 1:** The ChemProt corpus semantic relations

**Relation class**	**Eval.**	**ChemProt relations**
CPR:1	N	Part of
CPR:2	N	Regulator
CPR:3	Y	Upregulator and activator
CPR:4	Y	Downregulator and inhibitor
CPR:5	Y	Agonist
CPR:6	Y	Antagonist
CPR:7	N	Modulator
CPR:8	N	Cofactor
CPR:9	Y	Substrate and product of
CPR:10	N	Not

**Table 2:** The statistics of the ChemProt corpus

**Dataset**	**Abstracts**	**Chemicals**	**Genes/proteins**	**Evaluated CPIs**
Training	1020	13 017	12 735	4157
Development	612	8004	7563	2416
Test	3399	10 810	10 018	3458
Total	5031	31 813	30 316	10 031

**Figure 2:**
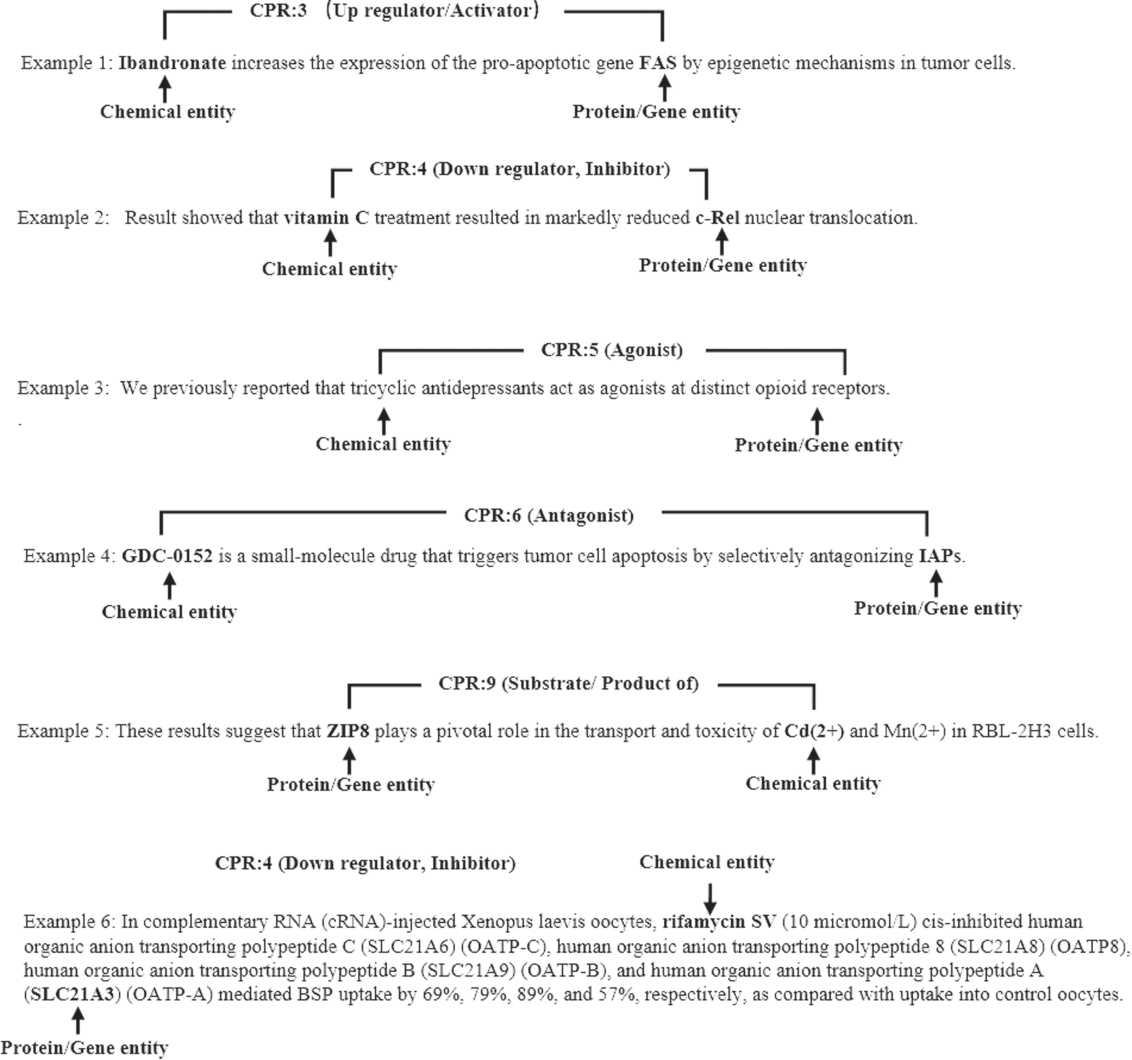
The illustrative examples of CPR classes.

Figure 2 gives some illustrative examples of CPI extraction. In example 1, ‘Ibandronate’ and ‘FAS’ (presented in bold) are chemical and gene entities, respectively. To accurately extract the CPI from example 1, we need not only to detect the interaction between ‘Ibandronate’ and ‘FAS’ but to also classify the interaction as the ‘CPR:3’ class. There are some long and complicated sentences in ChemProt corpus. Example 6 is a long sentence instance that contains multiple subsentences.

### The model architecture

**Figure 3:**
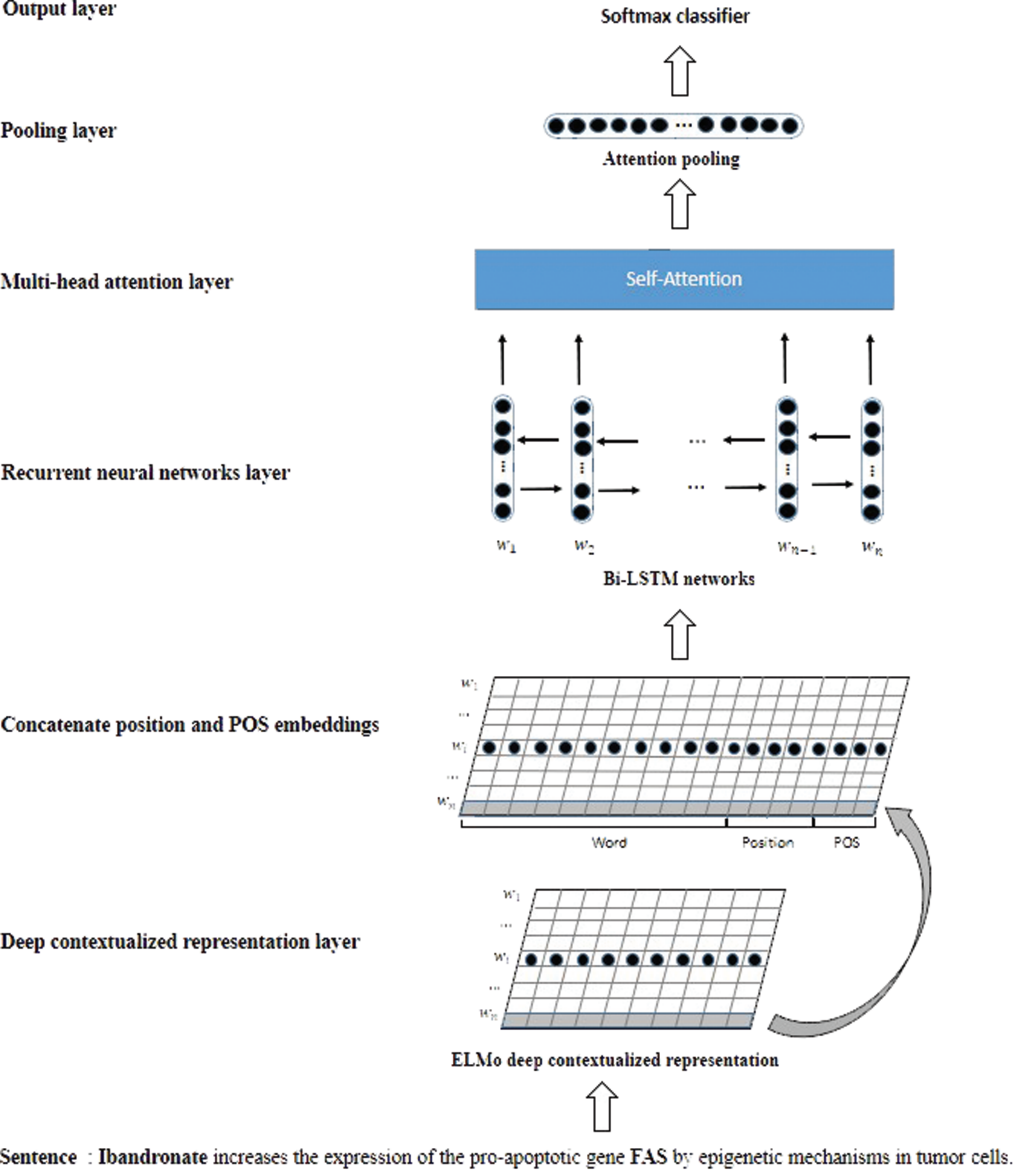
The schematic overview of our model.

Figure 3 is a schematic overview of our model. In a nutshell, our model mainly includes three parts: the deep contextualized representation layer, the Bi-LSTMs layer and the multihead attention layer. The inputs of our model are sentence sequences. The deep contextualized representation layer will generate the contextualized representation vector for each word based on the sentence context. Some recent studies (8, 24) have suggested that the position and part of speech (POS) of each word in the sentence are crucial to biomedical relation extraction. Hence, the word contextualized representation is concatenated with position and POS embeddings. The Bi-LSTMs layer will learn the latent features based on the whole word representations. The multihead attention layer applies a self-attention mechanism to capture the relative important features in the CPI extraction. After the multihead attention layer, we employed attention pooling and a softmax function to implement the detection and classification of the candidate CPIs in the sentences. In the following section, our CPI extraction model will be described in detail.

### Contextualized word representations

In our model, we used deep contextualized word (ELMo) representations instead of the traditional word embeddings. Unlike word embeddings, ELMo representations are functions of the input sentences based on bidirectional language models. Therefore, ELMo has the ability to generate different representation vectors for the same word according to the sentence context.

Given a sentence *S*, $\{{w}_1,{w}_2,\dots, {w}_n\}$ denotes the word sequence in the sentence. Given a word ${w}_k$, the forward language model calculates the probability of the word ${w}_k$ based on the front words $\{{w}_1,{w}_2,\dots, {w}_{k-1}\}$ of ${w}_k$ in *S* as follows:

(1) \begin{equation*} {P}_{forward}\!\left({w}_1,{w}_2,\dots, {w}_n\right)\!=\!{\prod}_{k=1}^np\!\left({w}_k|{w}_1,\!{w}_2,\!\dots,\! {w}_{k-1}\!\right).\end{equation*}

Similarly, the backward language model calculates the probability of the word ${w}_k$ based on the behind words $\{{w}_{k+1},{w}_{k+2},\dots, {w}_n\}$ of ${w}_k$ in *S* as follows:

(2) \begin{align*} &{P}_{backward}\left({w}_1,{w}_2,\dots, {w}_n\right)\nonumber\\&={\prod}_{k=1}^np\left({w}_k|{w}_{k+1},{w}_{k+2},\dots, {w}_n\right). \end{align*}

A bidirectional language model combines the forward and backward language models and jointly maximizes the log likelihood as follows:

(3) \begin{align*} &{\sum}_{k=1}^n\left( logp\left({w}_k|{w}_1,{w}_2,\dots, {w}_{k-1}\right)\right.\nonumber\\&+\left. logp\left({w}_k|{w}_{k+1},{w}_{k+2},\dots, {w}_n\right)\right). \end{align*}

ELMo representation is a function of a combination of the intermediate layer representations in the bidirectional language model. Therefore, ELMo can learn the different representation vector of each word in different sentences. More details about ELMo can be found in the study (18). In our experiments, we employ the ELMo module from TensorFlow Hub.

### Bi-LSTMs model

The LSTM model is currently one of the most powerful RNN models, which has been successfully applied in many NLP. Compared with traditional RNN models, the LSTM model employs the gate mechanism to solve the vanishing gradient problem (25). The LSTM model is a time sequential model and is explicitly designed to remember the information for long time periods. Therefore, the LSTM model is suitable to capture the long-term dependency feature in NLP tasks. At the time step *t*, each LSTM unit calculates the input word ${x}_t$, the previous hidden state ${h}_{t-1}$ and the memory cell ${c}_{t-1}$ to generate the current hidden state ${h}_t$ and memory cell ${c}_t$ (25). The current hidden state ${h}_t$ and memory cell ${c}_t$ can be calculated based on the equations (4)~(9). ${W}_{\ast }$, ${U}_{\ast }$ and ${b}_{\ast }$ denote weight and bias parameters of the LSTM units, and $\odot$ denotes element-wise multiplication. ${f}_t$ and ${o}_t$ are the state of the current forget and output gate, respectively.

(4) \begin{equation*} {f}_t= sigmoid\left({W}_f{x}_t+{U}_f{h}_{t-1}+{b}_f\right)\end{equation*}

(5) \begin{equation*} {o}_t= sigmoid\left({W}_o{x}_t+{U}_o{h}_{t-1}+{b}_o\right) \end{equation*}

(6) \begin{equation*} {g}_t=\mathit{\tanh}\left({W}_g{x}_t+{U}_g{h}_{t-1}+{b}_g\right) \end{equation*}

(7) \begin{equation*} {i}_t= sigmoid\left({W}_i{x}_t+{U}_i{h}_{t-1}+{b}_i\right) \end{equation*}

(8) \begin{equation*} {c}_t={f}_t\odot {c}_{t-1}+{i}_t\odot {g}_t\end{equation*}

(9) \begin{equation*} {h}_t={o}_t\odot \mathit{\tanh}\left({c}_{t-1}\right)\end{equation*}

The Bi-LSTMs model combines the forward LSTM and backward LSTM. Given${h}_t^f$ and ${h}_t^b$ denote the hidden state of the forward LSTM and backward LSTM, the final hidden state of the Bi-LSTMs will be concatenated into ${h}_t$=${h}_t^f\ \Big\Vert {h}_t^b$. The Bi-LSTMs model can deal with the input sequence from the two-way approach and capture more comprehensive features.

### Multihead attention

The Bi-LSTMs layer can effectively and automatically learn the latent features from the input sequences. However, only a small part of these latent features are crucial in the CPI extraction. In our model, multihead attention (21) is employed to further emphasize the relatively important features by adjusting the weights. The intuition behind the multihead attention is that applying the attention multiple times may learn more important features than single attention. In short, the attention mechanism calculates the output based on the query and a set of key–value pairs. The multihead attention is also based on query, key and value matrixes that are denoted as $Q,K,V\in {\mathbb{R}}^{n\times d}$. In our study, we used the multihead self-attention to deal with the output of Bi-LSTMs. The self-attention is a special case of multihead attention, which only requires a single input.

Given $X\in {\mathbb{R}}^{n\times d}$ denotes the input sequence matrix, the $Q,\kern0.5em K$ and $V$ will be generated by applying linear projections. Instead of the standard additive attention mechanism (26), the multihead attention uses dot-product attention to increase the parallel computation as follows:

(10) \begin{equation*} Attention\left(Q,K,V\right)= softmax\left(Q{K}^T/\sqrt{d}\right)V \end{equation*}

where $\sqrt{d}$ is the scaling factor. The key point of the multihead attention is employing the above attention multiple times. If the multihead attention contains $h$ heads, the *k*-th head attention${M}_k$ can be calculated as follows:

(11) \begin{equation*} {M}_k\left(Q,K,V\right)= Attention\left(Q{W}^Q,K{W}^K,V{W}^V\right) \end{equation*}

where ${W}^Q,{W}^K,{W}^V\in {\mathbb{R}}^{n\times d/h}$. The final multihead attention $M$ is the concatenation of $\{{M}_1,{M}_2,\dots, {M}_h\}$.

(12) \begin{equation*} M\left(Q,K,V\right)= Concat\left({M}_1,{M}_2,\dots, {M}_h\right){W}^O \end{equation*}

where ${W}^O\in {\mathbb{R}}^{d\times d}$. Thus, the output of the multihead attention $M(Q,K,V)$ is a matrix of ${\mathbb{R}}^{n\times d}$.

### Classification and training

In the pooling layer, the attention pooling (22) is applied to map the multihead attention matrix to the sentence vector representation. The ‘softmax’ function is used in the output layer to implement the detection and classification of CPIs.

In the experiments, our model is implemented by the Keras with the TensorFlow backend. We chose the categorical cross-entropy as the object function and use RMSProp to optimize the proposed model. A dropout mechanism was employed before the contextualized representation layer and output layer to alleviate the overfitting of the neural networks model (27). The hyperparameters used in our experiments are listed as follows. The hidden unit number of forward and backward LSTM is both 300. The mini-batch size is set as 64. The learning rate of RMSProp is set as 0.001. The dropout rate before the embedding layer and output layer is both set as 0.5. The early stopping strategy (28) is used to choose the number of epochs on the validation set.

## Results and discussion

### Datasets and evaluation metrics

The ChemProt corpus (13) is the major dataset for CPI extraction, which was released on the BioCreative VI share task. In Table 1, the ChemProt corpus is annotated for 10 relation classes, but only 5 relation classes (CPR:3, CPR:4, CPR:5, CPR:6 and CPR:9) were evaluated. To maintain the same evaluation with other studies, we also focused on the five-type relation classes. The statistics of the ChemProt corpus are listed in Table 2.

In the experiments, we combined the training and development sets as a whole training set. The validation set was randomly chosen from the training samples with a 10% rate. We used the validation to choose the parameters of our model. The test set was just used for evaluating the performance of our model.

The *F*-score, ‘precision’ and ‘recall’ are widely used as the evaluation metrics in CPI extraction. In particular, the *F*-score is the harmonic mean of both ‘precision’ and ‘recall’, which can quantify the overall performance. Therefore, the *F*-score was chosen as the major metric in our experiments. Since the CPI extraction is a multiclass classification task, we compute the micro average to assess the overall performance (14). To reduce the potential bias, we repeated each experiment five times and reported the average *F*-score, ‘precision’ and ‘recall’.

In the comparison experiments, we used the pretrained biomedical word embedding (29), which is available at https://github.com/cambridgeltl/BioNLP-2016. For ELMo model, we used pretrained ELMo embeddings from TensorFlow hub (https://tfhub.dev/google/elmo).

### Experimental results on ChemProt corpus

We first evaluated the effectiveness of different input representations of our method. In this experiment, we used the same Bi-LSTMs and changed the input representations. The comparison performance of different input representations are listed in Table 3. To focus on input representation evaluation, all models in Table 3 do not use attention strategy.

- ‘Word’: the input representation of the model is word embedding.
- ‘Word + Position’: the input representation of the model is the concatenated of word embedding and position embedding.
- ‘Word + Position + POS’: the input representation of the model is the concatenated of word embedding, position embedding and POS embedding.
- ‘Context’: the input representation of the model is deep context embedding.
- ‘Context + Position’: the input representation of the model is the concatenated of deep context embedding and position embedding.
- ‘Context + Position + POS’: the input representation of the model is the concatenated of deep context embedding, position embedding and POS embedding.

**Table 3:** The effect of the input representation on performance

**Input representation**	**Train vs validation**			**Train vs test**		
	**Precision**	**Recall**	***F*-score**	**Precision**	**Recall**	***F*-score**
Word	0.554	0.585	0.569	0.492	0.624	0.55
Word + Position	0.524	0.633	0.573	0.511	0.643	0.569
Word + Position + POS	0.601	0.596	0.598	0.586	0.57	0.578
Context	0.592	0.641	0.616	0.596	0.628	0.611
Context + Position	0.627	0.643	0.635	0.578	0.677	0.624
Context + Position + POS	0.629	0.66	0.644	0.621	0.64	0.629

Notes: ‘Word’, ‘Position’, ‘POS’ and ‘Context’ denote word embedding, position embedding, POS embedding and deep context representation, respectively. ‘Train vs validation’ and ‘Train vs test’ denote the results on validation and test sets, respectively. Attention mechanism is not used in this experiment.

**Table 4:** The effect of the attention heads and the dimensions on performance

**Heads**	**Dimensions**	**Train vs validation**			**Train vs test**		
		**Precision**	**Recall**	***F*-score**	**Precision**	**Recall**	***F*-score**
2	300	0.692	0.619	0.653	0.663	0.634	0.648
4	150	0.685	0.634	0.659	0.691	0.62	0.653
6	100	0.697	0.642	0.668	0.706	0.618	0.659
12	50	0.69	0.651	0.67	0.696	0.622	0.657
20	30	0.674	0.648	0.661	0.67	0.629	0.649

Notes: ‘Heads’ and ‘Dimensions’ denote the number of heads and the dimensions of $Q,$$K$ and $V$, respectively. ‘Train vs validation’ and ‘Train vs test’ denote the results on validation and test sets, respectively. The input representation is the combination of deep context embedding, position embedding and POS embedding.

**Figure 4:**
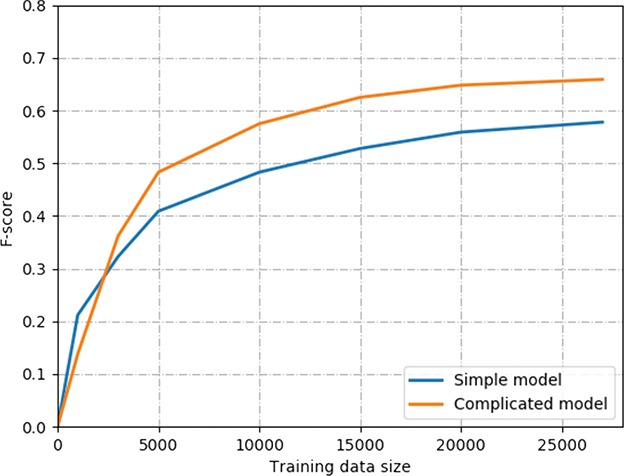
The learning curve of two models on ChemProt corpus. The simple model contains the combination input of the word, position and POS embeddings and Bi-LSTMs. The complicated model contains the combination input of deep context, position and POS embeddings, Bi-LSTMs and multihead attention mechanism.

In Table 3, it can be seen that the Bi-LSTMs model achieved an *F*-score of 0.55 when only using pretrained word embedding as the input representation. Both position embedding and POS embedding are helpful to improve the performance in CPI extraction. When combining the word, position and POS embedding, the *F*-score was improved from 0.55 to 0.578. Compared to the word embedding, deep context representation ELMo greatly improved the *F*-score from 0.578 to 0.629. The results suggest that the ELMo can generate a more comprehensive representation of the words from the intermediate layer based on the sentence contexts. This makes the ELMo outperform pretrained word embedding for the performance of CPI extraction. Moreover, combining the position and POS embeddings with deep context representation can further improve the performance. Overfitting is a common issue in this domain. We also report the performance measures on the validation set. Compared with the results on the validation set, the *F*-score only slightly decrease (≤0.015). This indicates that the models do not suffer much from overfitting.

Then, we evaluated the effectiveness of the multihead attention in CPI extraction. In this experiment, all models employed the multihead attention mechanism and used the concatenated of word embedding, position embedding and POS embedding as the input representation. Since multihead self-attention was employed, the dimensions of the query $Q,$ key $K$ and value $V$ are the same. As shown in Table 4, we varied the number of attention heads and the dimensions of $Q,$$K$ and $V$. In Table 4, we can see multihead attention can effectively improve the performance of CPI extraction. The experimental results indicate that multihead attention can combine the important features from the different heads to construct a comprehensive feature representation. We also noticed that the *F*-score ranged from 0.646 to 0.659 when setting different head numbers. When the number of heads is set too small or too large, the performance will drop off. Overall, our model achieved the highest *F*-score of 0.659 when the number of heads was set as 6, which outperformed the *F*-score of 0.629 without using multihead attention.

Next, we evaluated the performance with different sizes of training data. Generally, more labeled training samples are always helpful for the supervised learning model. However, in the biomedical domain, annotating labeled data is expensive and time-consuming. The key point is how many training data are required for competitive performance on biomedical relation extraction. Since we combined the training and development sets of the ChemProt corpus as a whole training set, the labeled training samples are ~27 000. We experimented with the differently labeled training samples that ranged from 1000 to 27 071. The evaluation results of the two models are shown in Figure 4. The simple model contains the combination input of the word, position and POS embeddings and Bi-LSTMs. The complicated model contains the combination input of deep context, position and POS embeddings, Bi-LSTMs and multihead attention mechanism. For the complicated model, the training sample size ranges from 1000 to 27 071, with a corresponding *F*-score of 0.128 to 0.659. Similarly, the corresponding performance of the simple model was increased from 0.222 to 0.578 in *F*-score. We made the observation that the performance increase slowed down when the number of training samples increased. For example, the corresponding performance of the complicated model increased from 0.128 to 0.483 when the training sample size increased from 1000 to 5000. However, the performance increase of the complicated model is only 0.011 when the training sample size increases from 20 000 to 27 071. Similar trends can be found for the simple model. Our results show that both models with ~ 5000 training samples can achieve >70% of the best performance. In addition, we can see the simple model outperforms the complicated model in *F*-score when using 1000 or 3000 training samples. The main reason is that the complicated model has much more parameters need to train than the simple model because of the ELMo representation and multihead attention layers. Therefore, the complicated model requires more training samples to train than the simple model. When the number of training samples is >5000, the complicated model can outperform the simple model significantly.

### Analysis of the word-level attention weights

**Figure 5:**
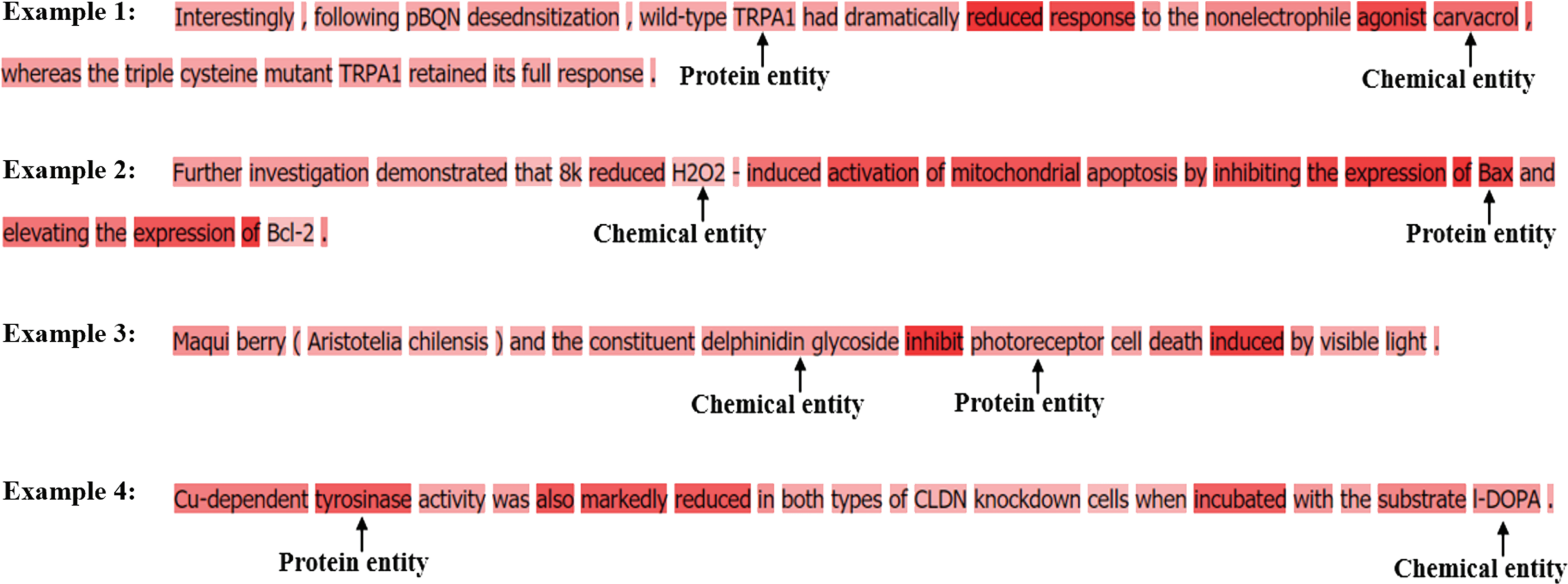
Examples of attention weight distribution.

Figure 5 shows the attention weight distribution of some instances from ChemProt test set. Darker background color (dark red) on the word indicates higher attention weights. It can be seen that the attention mechanism can highlight some important keywords, such as ‘agonist’, ‘inhibiting’ and ‘reduced’. These keywords are greatly helpful to classify the semantic relation type.

Table 5 gives the top 5 attention keywords in each relation type among the ChemProt test set. The recent study (22) has suggested that CPR:4, CPR:5 and CPR:6 have high concentration of keywords. We also found similar results. In Table 5, we can see, in CPR:4 (downregulator and inhibitor), all the top 4 key words are the variations of ‘inhibitor’. In CPR:5 (agonist) and CPR:6 (antagonist), the top words concentrate in the variation of ‘agonist’ and ‘antagonist’, respectively. These top words such as ‘inhibitor’, ‘agonist’ and ‘antagonist’ are the strong indicator of CPR:4, CPR:5 and CPR:6. For CPR:3 (upregulator and activator) and CPR:9 (substrate and product of), our model identified deeper semantic variants such as ‘increased’, ‘expression’ and ‘enzyme’.

**Table 5:** Top 5 attention keywords in each relation type

**CPR:3 (upregulator and activator)**	**CPR:4 (downregulator and inhibitor)**	**CPR:5 (agonist)**	**CPR:6 (antagonist)**	**CPR:9 (substrate and product of)**
Expression	Inhibitor	Agonist	Antagonist	Metabolism
Increased	Inhibition	Selective	Antagonists	Catalyzes
Induced	Inhibitors	Activity	Selective	Metabolized
Activation	Inhibited	Agonists	Receptor	Uptake
Activity	Cells	Antagonist	Binding	Enzyme

### Performance comparison on ChemProt corpus

We compared our method with other state-of-the-art methods on ChemProt corpus in Table 6. Warikoo *et al.* (30) employed a linguistic interaction pattern learning method to capture the CPI and achieved an *F*-score of 0.526 on ChemProt corpus. Lung *et al.* (31) used a three-stage model to integrate the semantic and dependency graph features and achieved an *F*-score of 0.567. Liu *et al.* (22) applied the GRU model with attention pooling and achieved an *F*-score of 0.527. Similarly, Corbett *et al.* (15) also used a Bi-LSTMs model with pretrained LSTM layers to achieve a high *F*-score of 0.615. Peng *et al.* (14) proposed an ensemble method to combine three system results including SVM, CNN and Bi-LSTMs. This method achieved an *F*-score of 0.641, which was the top rank in the BioCreative VI ChemProt share task.

**Table 6:** Performance comparison with other methods on ChemProt corpus

**Methods**	**Precision**	**Recall**	***F*-score**
Warikoo *et al.* (30)	0.592	0.474	0.526
Lung *et al.* (31)	0.632	0.512	0.567
Liu *et al.* (22)	0.574	0.487	0.527
Corbett *et al.* (15)	0.561	0.678	0.615
Peng *et al.* (14)	0.727	0.574	0.641
Our method	0.706	0.618	0.659

Notes: the input representation is the combination of deep context embedding, position embedding and POS embedding. The number of the attention heads and dimensions are set as 6 and 100, respectively.

In Table 6, neural network-based methods achieved highly competitive performance in the CPI extraction task. Compared with other methods, our model effectively integrated the deep context representation, Bi-LSTMs and multihead attention and achieved the highest *F*-score of 0.659. We noticed that some studies (14, 15, 22) also applied Bi-LSTMs in CPI extraction. The Bi-LSTMs model can automatically learn the long-term latent features from the sentence. For CPI extraction, some sentences are long and complicated. Moreover, some chemical and protein entities are in different clauses. Thus, it is difficult to learn enough features for Bi-LSTMs to distinguish and classify the candidate CPI. To solve this problem, we applied the deep context representation instead of word embedding to generate the comprehensive sentence representation, which provided much more comprehensive information to the Bi-LSTMs. Furthermore, we employed a multihead attention layer to effectively enhance the important distinguishing features based on the Bi-LSTMs output. Therefore, both the deep context representation and multihead attention strategies were helpful to improve the performance in CPI extraction. Our experiments were performed on NVIDIA GPU GeForce GTX Titan Xp with 12 GB GDDR5X memory. The training time for one epoch of our model was ~317 s.

Overall, our method took advantage of the deep context representation and multihead attention strategies to achieve state-of-the-art performance on the ChemProt corpus.

### Performance breakdown and error analysis

Table 7 gives the performance breakdown of each CPI type. It can be seen that the performance of different CPI type vary significantly. Our model achieved a high *F*-score of 0.725 and 0.718 on CPR:6 (antagonist) and CPR:4 (downregulator and inhibitor), respectively. On the contrary, the *F*-score on CPR:9 (substrate and product of) is only 0.501. This indicates that it is the most difficult for our model to accurately classify CPR:9 type CPIs. Table 8 shows the confusion matrix for our model on the test set. The *x*-axis is the predicted label by our model, and the *y*-axis is the gold standard label. In Table 8, we can see that the major challenge is nonrelations being mistaken for relations and vice versa. Besides that, there is something of a problem with CPR:3 (upregulator and activator) being mistaken for CPR:4 (downregulator and inhibitor). This suggests that accurately distinguishing between CPR:3 and CPR:4 is another challenge for our model.

**Table 7:** Performance breakdown of our model on the test set

**Label**	**Support**	**Precision**	**Recall**	***F*-score**
CPR:3	664	0.662	0.539	0.594
CPR:4	1661	0.704	0.732	0.718
CPR:5	194	0.737	0.593	0.657
CPR:6	281	0.759	0.694	0.725
CPR:9	643	0.735	0.379	0.501

Notes: the input representation is the combination of deep context embedding, position embedding and POS embedding. The number of attention heads and dimensions are set as 6 and 100, respectively.

**Table 8:** Confusion matrix for our model on the test set

**Gold**	**False**	**CPR:3**	**CPR:4**	**CPR:5**	**CPR:6**	**CPR:9**
False	10 267	164	415	32	51	84
CPR:3	220	358	80	3	2	1
CPR:4	427	14	1216	0	1	3
CPR:5	66	2	3	115	8	0
CPR:6	77	0	3	6	195	0
CPR:9	386	3	10	0	0	244

Notes: the input representation is the combination of deep context embedding, position embedding and POS embedding. The number of attention heads and dimensions are set as 6 and 100, respectively.

**Figure 6:**
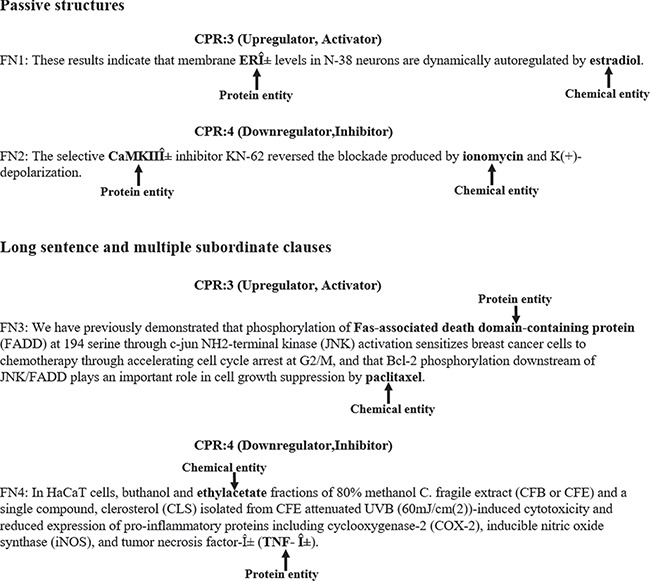
Examples of false negatives.

In addition, we also manually analyzed what sentences lead to false negatives. In Figure 6, we gave some examples of false negatives. The chemical and protein entities are in bold. We noted that the passive structure is a frequent cause of false negatives. For example, in FN1, the chemical entity ‘estradiol’ is in passive. In this case, our model misclassified the relation between ‘ERÎ±’ and ‘estradiol’ as false. Another frequent cause of false negatives is the sentence is long and complicated. For example, in FN3, the chemical and protein entities are in different clauses and the sentence is relatively complicated. In this case, our model failed in identifying the relation between ‘Fas-associated death domain-containing protein’ and ‘paclitaxel’. In the future plan, more effort should be made on how to identify CPIs from passive structures and long sentences accurately.

### Experimental results on DDI 2013 corpus

We also evaluated our model on DDI 2013 corpus (6, 32) that contains four DDI types: ‘Advice’, ‘Effect’, ‘Mechanism’ and ‘Int’. Table 9 shows the detailed statistics of DDI 2013 corpus. In this experiment, we used the same hyperparameters. Since the recent studies (8, 24) have suggested that the position and POS embedding can improve the performance of DDI extraction, we concatenated the word or deep context embedding with position and POS embedding as the input representation in this experiment. We mainly focused on the effectiveness of context representation and multihead attention strategy in the ablation study. Table 10 gives the ablation study results on the DDI 2013.

- ‘Word + Position + POS’: the input representation of the model is the concatenated of word embedding, position embedding and POS embedding. Attention mechanism is not used in this model.
- ‘Context + Position + POS’: the input representation of the model is the concatenated of deep context embedding, position embedding and POS embedding. Attention mechanism is not used in this model.
- ‘Context + Position + POS + Attention’: the input representation of the model is the concatenated of deep context embedding and position embedding. The multihead attention strategy is employed in this model. The number of attention heads and dimensions are set as 6 and 100, respectively.

**Table 9:** The statistics of the DDI extraction 2013 corpus

**Corpus**	**Advice**	**Effect**	**Mechanism**	**Int**	**Negative**
Training set	826	1687	1319	188	23 772
Test set	221	360	302	96	4737
Total	1047	2047	1621	284	28 554

**Table 10:** The ablation study on DDI 2013 corpus

**Models**	**Precision**	**Recall**	***F*-score**
Word + Position + POS	0.774	0.62	0.688
Context + Position + POS	0.787	0.658	0.716
Context + Position + POS + Attention	0.782	0.674	0.724

Notes: ‘Word’, ‘Position’, ‘POS’, ‘Context’ and ‘Attention’ denote word embedding, position embedding, POS embedding, deep context representation and attention mechanism, respectively.

In Table 10, it can be seen that both context representation and multihead attention strategy are also helpful on DDI 2013 corpus. In particular, compared to word embedding, the context representation embedding improved the *F*-score from 0.688 to 0.716.

In Table 11, we compared our model with other state-of-the-art methods on DDI 2013 corpus. Zhao *et al.* (8) employed CNN model to extract DDIs and achieved an *F*-score of 0.686. Raihani and Laachfoubi (33) constructed rich features and employed feature-based method to extract DDIs and achieved a high *F*-score of 0.711. Quan *et al.* (34) used multichannel CNN model to extract DDIs, which can effectively integrate multiple input representation for DDI extraction task. Sahu and Anand (35) applied Bi-LSTM model and attention pooling to extract DDIs, which achieved a high *F*-score of 0.711. Zhang *et al.* (12) proposed a hybrid model to combine CNNs and Bi-LSTM for DDI extraction task. To boost the performance, the hybrid model input not only the token sequence of sentences but also the shortest dependency path between the two drug entities. The hybrid model achieved the highest *F*-score of 0.737 on DDI 2013 corpus. All these methods focused on the DDI extraction task. Compared with other methods, our model can achieve competitive performance on DDI 2013 without fine-tuning hypermeters. This indicated that our model can be applied to other biomedical relation extraction task.

**Table 11:** Performance comparison with other methods on DDI 2013 corpus

**Methods**	**Precision**	**Recall**	***F*-score**
Zhao *et al.* (8)	0.725	0.651	0.686
Raihani and Laachfoubi (33)	0.737	0.687	0.711
Quan *et al.* (34)	0.76	0.653	0.702
Sahu and Anand (35)	0.734	0.697	0.715
Zhang *et al.* (12)	0.75	0.725	0.737
Our method	0.782	0.674	0.724

Notes: the input representation is the combination of deep context embedding, position embedding and POS embedding. The number of attention heads and dimensions are set as 6 and 100, respectively.

## Conclusion

Accurately detecting and extracting CPIs from the literature is a crucial task in the biomedical domain. However, the best performance of CPI extraction is ~0.64 in *F*-score. Both the deep context representation and multihead attention strategies are the most recent advantages of deep learning, which could improve the performance of CPI extraction. The deep context representation can effectively generate the sentence representation according to the sentence contexts. The multihead attention mechanism learns the important features from different heads and has the ability of generating more comprehensive and distinguished feature representation. In this work, we proposed a neural networks-based method to integrate the deep context representation, Bi-LSTMs and multihead attention in CPI extraction. The proposed method was evaluated on the recent ChemProt corpus. The results show that both deep context representation and multihead attention improve the performance in CPI extraction. It is encouraging to see that the proposed model achieved the highest performance of 0.659 in *F*-score on the ChemProt corpus. The experimental results on DDI 2013 also suggest our methods can be applied to other biomedical relation extraction tasks.

Generally, supervised learning methods depend on sufficient labeled training data. However, annotating training data is expensive and time-consuming, especially so in biomedicine as domain knowledge is required. In the future, we will explore the effectiveness of semi-supervised learning or transfer learning in CPI extraction.

## Notes

**Database URL:** https://github.com/zhangyijia1979/CPI_extraction
